# Refractory thrombocytopenia induced by enzalutamide combined with docetaxel chemotherapy in a metastatic castration-resistant prostate cancer patient with bone marrow metastasis: a case report and literature review

**DOI:** 10.3389/fonc.2026.1863480

**Published:** 2026-08-31

**Authors:** Yong Chen, Junbo Liu

**Affiliations:** Department of Urology, West China School of Medicine, Sichuan University, Sichuan University Affiliated Chengdu Second People’s Hospital, Chengdu Second People’s Hospital, Chengdu, China

**Keywords:** bone marrow metastasis, docetaxel, enzalutamide, metastatic castration-resistant prostate cancer (mCRPC), refractory thrombocytopenia

## Abstract

**Background:**

Enzalutamide combined with docetaxel is one of the effective therapeutic regimens for metastatic castration-resistant prostate cancer (mCRPC), which significantly prolongs progression-free survival (PFS). For mCRPC patients with bone marrow metastasis (BMM), bone marrow metastasis itself impairs the bone marrow microenvironment and results in reduced hematopoietic function. The combined therapy may induce life-threatening refractory thrombocytopenia, which has been rarely reported in clinical practice.

**Case presentation:**

A 62-year-old male patient with mCRPC and confirmed bone marrow metastasis was reported herein. After progression to mCRPC, the patient was treated with enzalutamide plus docetaxel, followed by grade 4 refractory thrombocytopenia. The lowest platelet count decreased to 3×10^9^/L, and the patient showed poor response to conventional supportive care. Bone marrow biopsy confirmed progressive infiltration of tumor cells in the bone marrow with occasional megakaryocytes. Docetaxel and enzalutamide were discontinued, and platelet transfusion and subcutaneous injection of recombinant human thrombopoietin were administered. The hemorrhagic manifestations were relieved, while platelet count remained persistently low.

**Conclusion:**

Enzalutamide combined with docetaxel may increase the risk of refractory grade 4 thrombocytopenia among mCRPC patients with bone marrow infiltration, as illustrated by this single clinical case.

## 1 Introduction

Metastatic hormone-sensitive prostate cancer (mHSPC) is associated with poor prognosis once it progresses to metastatic castration-resistant prostate cancer (mCRPC). The PRESIDE study has demonstrated that enzalutamide combined with docetaxel significantly improves progression-free survival (PFS) in patients with mCRPC (1–3). In mCRPC patients complicated with bone marrow metastasis (BMM), tumor cell infiltration into hematopoietic tissues increases susceptibility to chemotherapy-induced bone marrow toxicity by disrupting the hematopoietic microenvironment (4–6). Moreover, docetaxel-induced myelosuppression is a well-recognized dose-limiting toxicity (7), and enzalutamide can also cause grade 3–4 hematological adverse reactions (1, 8). Previous reports have indicated that the combination of docetaxel with abiraterone, enzalutamide, or platinum agents increases the risk of myelosuppression, which mainly affects neutrophils and has minor impact on platelets (9–11). This paper reports a case of grade 4 refractory thrombocytopenia in an mCRPC patient with bone marrow metastasis after treatment with enzalutamide combined with docetaxel. We hypothesize that the dual myelosuppressive effects of docetaxel and enzalutamide may jointly suppress residual hematopoietic function, which represents a tentative inference based only on temporal clinical observation rather than confirmed causal synergy. Currently, there is no standardized management strategy for this clinical scenario, and few reports are available in real-world clinical practice. This study aims to investigate its clinical significance and management principles.

## 2 Case presentation

### 2.1 Patient data and baseline characteristics

A 62-year-old male patient underwent laparoscopic radical prostatectomy 2 years prior. Postoperative pathology revealed a Gleason score of 4 + 5 = 9 with intraductal carcinoma and bilateral seminal vesicle invasion. After 6 months of androgen deprivation therapy (ADT), total prostate-specific antigen (tPSA) decreased to a minimum of 0.2 ng/ml. The disease progressed to castration-resistant prostate cancer (CRPC) 17 months later, with a tPSA level of 267.05 ng/ml. At 20 months, positron emission tomography-computed tomography (PET-CT) was performed due to bone pain, indicating increased bone metabolism in multiple sites throughout the body, suggestive of extensive bone metastasis and progression to mCRPC.

- Past medical history: No history of primary hematological diseases, autoimmune diseases, or chronic hepatic and splenic dysfunction.
- Baseline laboratory examinations before treatment: tPSA 563.13 ng/mL, free prostate-specific antigen (fPSA) 68.94 ng/mL; platelet count 115×10^9^/L; white blood cell count 11.38×10^9^/L; red blood cell count 2.91×10¹²/L; hemoglobin 92 g/L; normal liver and renal function parameters.

### 2.2 Therapeutic regimen

In accordance with the 2024/2025 Chinese Society of Clinical Oncology (CSCO) Guidelines for the Diagnosis and Treatment of Prostate Cancer, the patient was prescribed enzalutamide 160 mg orally once daily, combined with docetaxel 75 mg/m² intravenously every 3 weeks, and prednisone 5 mg orally twice daily concurrently.

### 2.3 Clinical course of adverse events

**Figure 1:**
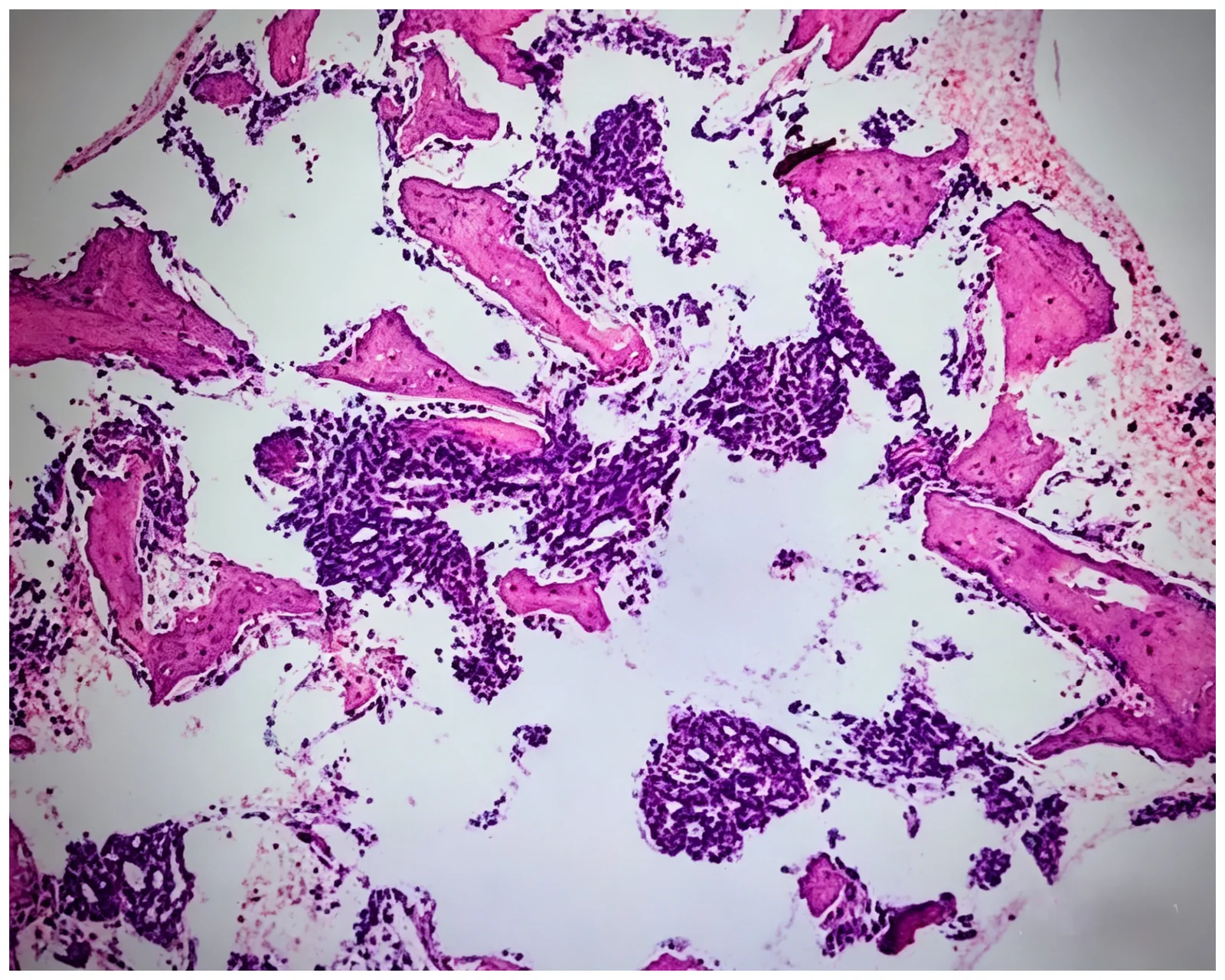
Bone marrow histology demonstrates metastatic prostate cancer infiltration and reduced megakaryocytes, resulting in refractory thrombocytopenia during enzalutamide and docetaxel combination treatment.

1. Onset and progression: After the first cycle of treatment, the patient’s platelet count began to decrease progressively on day 10 post-chemotherapy and developed grade 4 thrombocytopenia on day 15 post-chemotherapy, with platelet count dropping to 3×10^9^/L accompanied by gingival bleeding.
2. Initial management: Enzalutamide and docetaxel were discontinued immediately, and multiple platelet transfusions and subcutaneous injection of recombinant human thrombopoietin 15,000 U once daily were administered.
3. Refractory manifestations: After 14 days of intensive supportive care, the patient’s platelet count remained persistently below 20×10^9^/L (minimum 19×10^9^/L), meeting the diagnostic criteria for refractory thrombocytopenia.
4. Further examinations: Bone marrow biopsy confirmed disordered bone trabeculae, bone marrow fibrous hyperplasia, infiltration of atypical glands in the medullary cavity, scattered distribution of a small number of granulocytes and erythrocytes at various stages, and occasional megakaryocytes. Reticulin special staining revealed grade 2 myelofibrosis (MF-2). No quantitative counting data for tumor infiltration proportion or marrow cellularity was available in the pathological report. (**Figure 1**). Immunohistochemistry results: PCK(+), PSA(+), NKX3.1(+), CDX-2 (–), TTF-1 (–), MPO (–), CD235a (–), CD61 (–).
5. Differential diagnosis:D-dimer was mildly elevated against an otherwise unremarkable coagulation profile. Peripheral smear showed no schistocytes, LDH remained within normal limits, and renal function was preserved, effectively excluding DIC and TTP. Vitamin B12 (939 pg/mL) and folate (>20 ng/mL) were within normal range, ruling out deficiency-related cytopenia. Ferritin was markedly elevated at 10651 ng/mL, in keeping with underlying tumor-associated inflammation. Viral serologies were negative, excluding infection-associated thrombocytopenia. The patient was heparin-naïve with negative HPA/HLA alloantibody screening, rendering HIT unlikely. Bone marrow biopsy revealed diffuse infiltration by atypical prostatic glands accompanied by myelofibrosis, with paucity of granulocytic and erythroid precursors and markedly reduced megakaryocytes. The pattern of selective megakaryocyte depletion amid marrow replacement argues against immune-mediated thrombocytopenia as the dominant process and implicates combined drug- and tumor-related myelotoxicity.
6. Treatment course: Both docetaxel and enzalutamide were discontinued when persistent Grade 4 thrombocytopenia was developed. The patient received multiple platelet transfusions and daily subcutaneous recombinant human thrombopoietin for 17 days. Despite these supportive measures, platelet counts plateaued at 30–40×10^9^/L without further recovery, although hemorrhagic manifestations resolved completely. Progressive disease—rising PSA, extensive marrow-infiltrative bone metastases, and refractory bone pain—prompted consideration of further therapy. Enzalutamide monotherapy was initiated after discussion of available options, informed by its phase III profile in which severe cytopenias are uncommon (<1% Grade 3–4) (12)and by prior case reports describing rechallenge without recurrent severe thrombocytopenia (13). Following reintroduction, platelet counts remained stable at 30–40×10^9^/L with no recurrent bleeding. Four weeks after discharge, PSA declined from 398.39 to 260.13 ng/mL, with partial relief of bone pain. Notably, the absence of recurrent severe thrombocytopenia following enzalutamide rechallenge is consistent with—though does not prove—the interpretation that concurrent dual-agent exposure, rather than either agent alone, drove the initial refractory event.
7. Follow-up and clinical outcomes: Serial laboratory monitoring during follow-up showed incomplete recovery of platelet counts, and no new active bleeding events were recorded throughout the observation period.

### 2.4 Summary of clinical timeline

**Figure 2:**
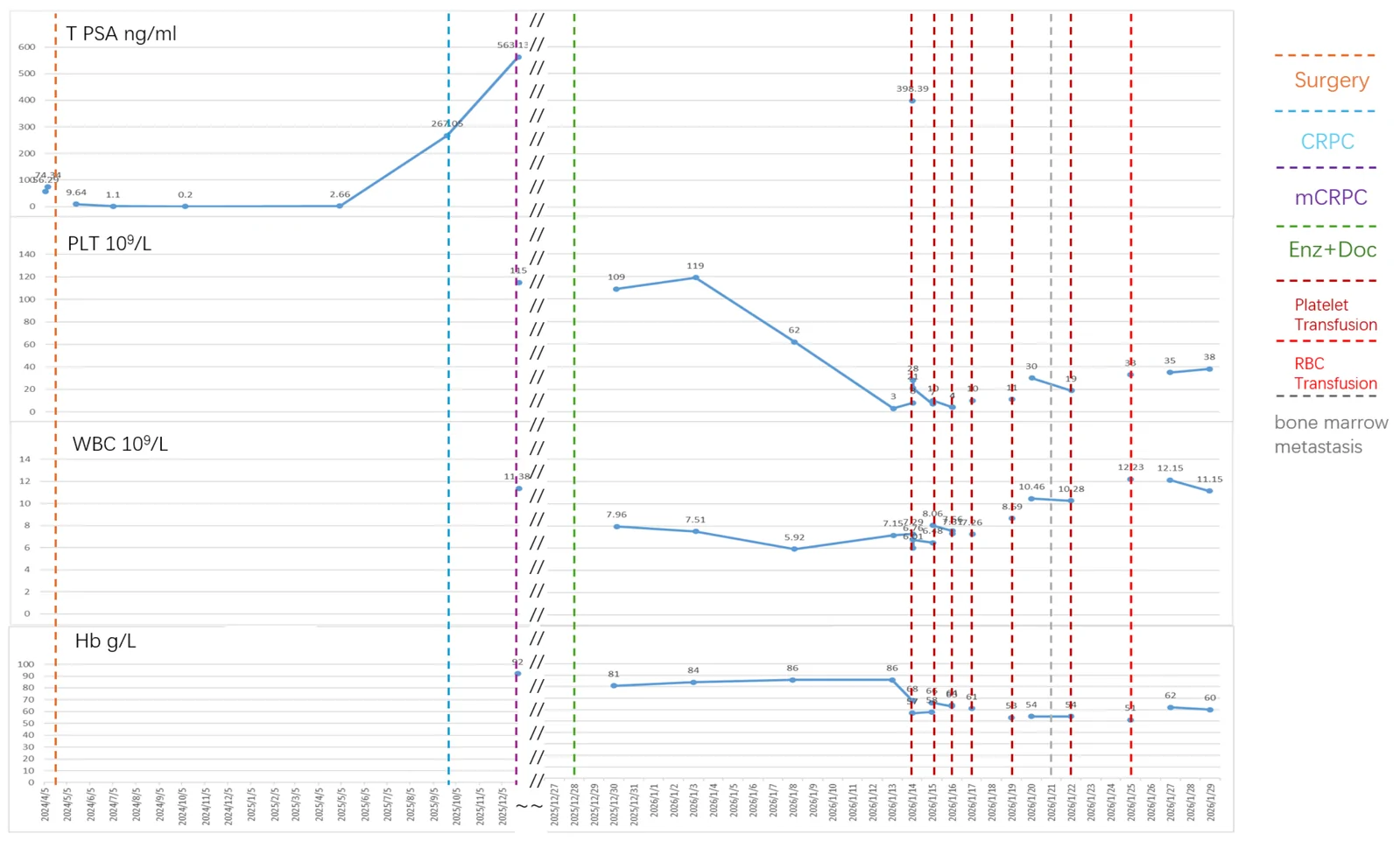
Presents the clinical timeline of this case, covering key time points including confirmation of bone marrow metastasis, initiation of treatment, onset of thrombocytopenia, implementation of interventions, and recovery of platelet count.

The clinical timeline of the patient is summarized in **Figure 2**.

## 3 Discussion

Chemotherapy is one of the effective therapeutic options for mCRPC patients, but it is usually accompanied by bone marrow suppression. The effects of taxane chemotherapeutic agents (docetaxel, cabazitaxel) on bone marrow are mainly concentrated on neutropenia, with an incidence of grade 3 or higher leukopenia reaching 41.7% (12). Thrombocytopenia, anemia, and lymphopenia are also common components of myelosuppression, but their incidences are much lower than that of neutropenia (13). The main causes of chemotherapy-induced thrombocytopenia are insufficient platelet production due to drug-induced inhibition of megakaryocytes and/or excessive platelet destruction caused by immune or non-immune factors (14). Thrombocytopenia can be corrected by standardized platelet transfusion and injection of recombinant human thrombopoietin (rhTPO)/recombinant human interleukin-11 (rhIL-11) (15–17).

The patient had mCRPC with extensive bone marrow infiltration and marked disruption of thehematopoietic microenvironment. Baseline platelet count measured 115 × 10^9^/L at the borderline low threshold; moderate anemia was present while granulocyte numbers remained intact, reflecting reduced marrow reserve capacity. After combination therapy was started, severe isolated thrombocytopenia emerged, with steady neutrophil levels and only a mild drop in hemoglobin values. Diffuse bone marrow tumor infiltration generally suppresses all three hematopoietic lineages simultaneously. The selective loss of megakaryocytes seen in this patient cannot be fully explained by metastatic involvement alone. We completed comprehensive laboratory testing, including peripheral blood smears, autoimmune antibody screening, viral assays, coagulation tests and nutritional indicators, to exclude ITP, HIT, DIC, TTP, infection-triggered thrombocytopenia and cytopenias secondary to nutritional deficiency. Together, the data in **Supplementary Table 1** (13, 18–20) indicate that docetaxel and enzalutamide each cause myelosuppression, and their combined effect may compound preexisting marrow damage while selectively suppressing megakaryopoiesis. This observation may partly account for the limited efficacy of standard supportive treatment. Still, we can only confirm a chronological correlation between treatment and thrombocytopenia; there is no experimental evidence to verify synergistic myelotoxicity between the two drugs (21). In clinical practice, extensive bone metastasis should alert clinicians to the possibility of bone marrow involvement (22). Findings from this case suggest that baseline bone marrow assessment—including biopsy where clinically indicated—may be considered in mCRPC patients prior to combined enzalutamide and docetaxel therapy. This evaluation can quantify tumor infiltration within bone marrow and measure remaining hematopoietic capacity, which helps clinicians adjust treatment order or drug dosages accordingly. This case raises consideration of intensified hematologic surveillance, such as twice- to thrice-weekly complete blood count testing, during combined enzalutamide and docetaxel treatment. If thrombocytopenia develops, providers might consider pausing the dual-agent combination promptly to lessen the risk of serious bleeding, with alternative treatment plans explored as needed. This case report is limited by its single-case design. Large-sample real-world cohort studies are needed in the future to clarify the actual incidence of such adverse events and explore the optimal clinical management strategies.

## 4 Patient perspective

I am a 62-year-old patient diagnosed with metastatic castration-resistant prostate cancer (mCRPC) with extensive bone metastasis and bone marrow metastasis, following radical prostatectomy and androgen deprivation therapy. I was treated with combination therapy of enzalutamide plus docetaxel. After the first cycle, I rapidly developed grade 4 refractory thrombocytopenia with bleeding manifestations, which was poorly responsive to conventional supportive care. Bone marrow biopsy confirmed extensive tumor infiltration and severely impaired hematopoiesis. Following discontinuation of the combination regimen, platelet transfusion, and administration of recombinant human thrombopoietin, bleeding was controlled, but platelet counts were not fully restored. Subsequent treatment was switched to enzalutamide monotherapy with intensive hematologic monitoring. My clinical course indicates that mCRPC patients complicated by bone marrow metastasis might face elevated risk of severe myelosuppression when receiving this dual-agent regimen; such observations call for personalized treatment plans and close hematologic monitoring on a case-by-case basis.

## 5 Conclusion

This case describes severe refractory thrombocytopenia complicating concurrent docetaxel and enzalutamide therapy in a patient with marrow-infiltrative mCRPC. Notably, stable platelet counts without recurrent severe thrombocytopenia following reintroduction of enzalutamide monotherapy suggest that combined dual-drug exposure—rather than either agent alone—was the likely driver of the severe megakaryocyte depletion observed on biopsy. Definitive causal links cannot be established based solely on this individual patient’s clinical course.

## Data Availability

The original contributions presented in the study are included in the article/**Supplementary Material**. Further inquiries can be directed to the corresponding author.

## Supplementary material

Supplementary Table 1 Comparative summary of thrombocytopenia induced by enzalutamide combined with docetaxel in patients with various solid malignancies.
